# Exceptional Response to Pembrolizumab in Metastatic ER+/HER2− Breast Cancer With Liver Metastases: A Case Report and Literature Review

**DOI:** 10.1155/crom/7970572

**Published:** 2025-10-14

**Authors:** Claudia Villa Celi, Supriya Peshin, Adit Dharia, Faizan Bashir, Linden Erica

**Affiliations:** ^1^Department of Internal Medicine, Capital Health Medical Center, Trenton, New Jersey, USA; ^2^Department of Internal Medicine, Norton Community Hospital, Norton, Virginia, USA; ^3^Department of Internal Medicine, HCA Florida Oak Hill Hospital, Brooksville, Florida, USA; ^4^School of Medicine, Shiraz University of Medical Sciences, Shiraz, Iran; ^5^Department of Oncology, Capital Health Medical Center, Trenton, New Jersey, USA

**Keywords:** breast cancer, hormone receptor–positive, immunotherapy, pembrolizumab

## Abstract

**Background:**

Hormone receptor–positive (HR+) and HER2-negative breast cancer is the most common subtype in women, particularly in the postmenopausal setting. Unlike triple-negative breast cancer, the benefit of immune checkpoint inhibitors (ICIs) in HR+/HER2− disease remains uncertain because of low tumor immunogenicity and limited PD-L1 expression.

**Case Presentation:**

We describe a case of a 70-year-old woman who presented with severe anemia and was incidentally found to have a bleeding left breast mass. Biopsy confirmed Grade 3 invasive ductal carcinoma (ER+/PR+ > 95%, HER2−) with nodal involvement but no distant metastases, consistent with Stage IIIc disease. She was treated with neoadjuvant anastrozole, modified radical mastectomy, adjuvant chemotherapy, radiation, and continued endocrine therapy. After 3 years, she developed extensive hepatic metastases. Biopsy revealed ER+/PR−/HER2− disease with striking PD-L1 expression (CPS 95%). The disease progressed on fulvestrant and palbociclib, but switching to carboplatin, gemcitabine, and pembrolizumab led to rapid improvement: liver function normalized and imaging showed near-complete response within 3 months. This remission lasted about 10 months before disease progression and transition to hospice care.

**Conclusion:**

This case explains the potential role of ICIs in HR+/HER2− breast cancer with unusually high PD-L1 expression. It underscores the importance of biomarker-driven treatment and supports expanding PD-L1 testing to better identify patients who may benefit from immunotherapy in this traditionally resistant subtype.

**Summary**

• Immune checkpoint inhibitors (ICIs) may have a role in hormone receptor–positive (HR+) and human epidermal growth factor receptor 2-negative (HER2−) metastatic breast cancer with high programmed death-ligand 1 (PD-L1) expression.
• The exceptional response to pembrolizumab underscores the need for future research into immunotherapy-driven approaches, potentially expanding treatment options beyond conventional endocrine and chemotherapy regimens.

## 1. Background

Breast cancer is the most frequently diagnosed cancer among women in the United States and North America, representing a significant public health burden [1, 2]. HR+ and HER2− breast cancers are the most common subtypes, accounting for approximately 60%–70% of all cases [3, 4]. Despite advancements in treatment, metastatic HR+/HER2− breast cancer remains challenging, with endocrine resistance often limiting long-term survival [5]. Current standard therapies, including endocrine therapy and cyclin-dependent kinase 4/6 (CDK4/6) inhibitors, have improved outcomes; however, the challenge of inevitable progression in advanced disease remains [6].

ICIs, which enhance the immune system's ability to detect and destroy cancer cells, have revolutionized the treatment of various malignancies [7]. However, their efficacy in HR+/HER2− breast cancer has been less pronounced compared to other subtypes, such as triple-negative breast cancer (TNBC) [8]. Lower PD-L1 expression and reduced immune infiltration in HR+ tumors are thought to contribute to this limited response [9]. Emerging evidence suggests that select HR+/HER2− breast cancer cases, particularly those with high tumor PD-L1 expression or significant tumor burden, may benefit from ICIs [10–12].

This report highlights the case of a 70-year-old woman with metastatic HR+/HER2− breast cancer and extensive liver metastases who demonstrated a remarkable response to pembrolizumab-based therapy. By exploring this case and reviewing relevant clinical trials, including the PACE trial and KEYNOTE-756 [13, 14], we aim to emphasize the potential role of ICIs in HR+ breast cancer and discuss future directions for integrating immunotherapy in this challenging disease subtype.

## 2. Case Presentation

A 70-year-old woman presented with severe anemia, with her hemoglobin level measuring 5.6 g/dL. She was initially evaluated for suspected peptic ulcer disease; however, during this work-up, an incidental finding of a large, ulcerated, and actively bleeding mass was noted in the left breast, specifically in the lower outer quadrant.

A core needle biopsy of the breast lesion revealed invasive ductal carcinoma (IDC), Grade 3. Immunohistochemistry showed strong expression of estrogen and progesterone receptors (> 95% positivity) and was negative for HER2. A biopsy of a regional lymph node confirmed metastatic involvement. Staging studies demonstrated no evidence of distant metastases, leading to a clinical diagnosis of Stage IIIc breast cancer (cT4a cN1).

**Figure 1:**
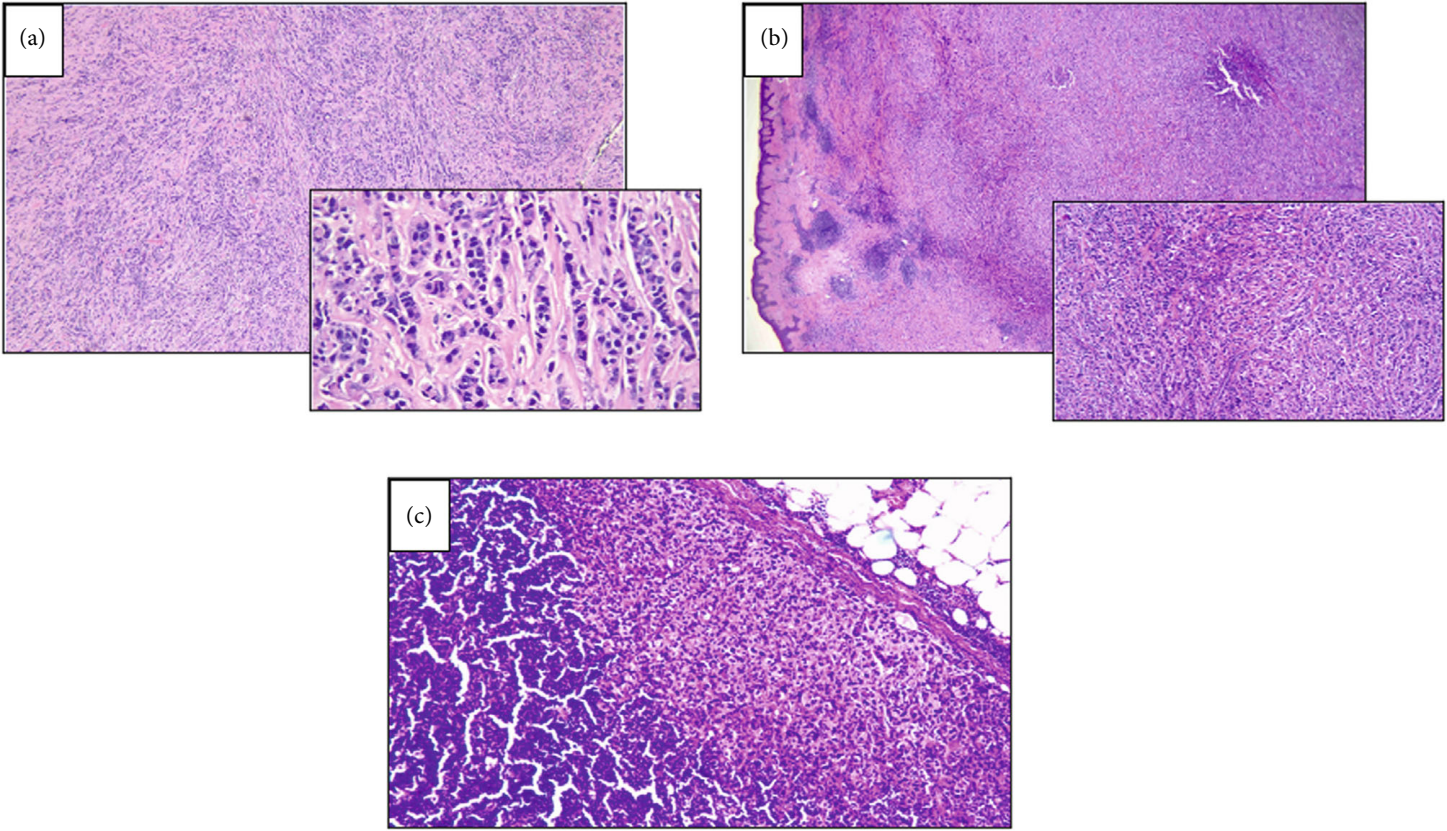
Histologic features of the tumor. Hematoxylin–eosin–stained sections of breast and lymph node demonstrating invasive infiltrating ductal carcinoma. (a) Breast cancer at the time of diagnosis (10x and 40x). (b) Breast cancer after neoadjuvant chemotherapy (NAC). (c) Lymph node after NAC.

The patient was started on anastrozole, which resulted in marked clinical improvement and near-complete resolution of the breast mass. Given this favorable response, she underwent a left modified radical mastectomy (MRM). Histopathological examination confirmed residual Grade 3 IDC with extensive lymphovascular invasion and involvement of one out of six lymph nodes (ypT2 ypN1) (Figure 1). Repeat staging with laboratory studies and PET imaging revealed no distant disease.

Postoperatively, she received dose-dense adriamycin and cyclophosphamide, followed by paclitaxel (ddAC-Taxol), in addition to comprehensive radiation therapy. She subsequently resumed endocrine therapy with anastrozole.

Three years later, at the age of 73, she developed biochemical evidence of disease recurrence, with a CA 27.29 level of 75.2 U/mL. PET imaging revealed multiple hepatic lesions, and a biopsy confirmed metastatic breast carcinoma (ER+, PR−, HER2− by IHC, and GATA3 positive). Next-generation sequencing (Foundation One) identified strikingly high PD-L1 expression (CPS 95%). This was an unusual finding in ER+/HER2− disease and suggested potential sensitivity to immunotherapy.

**Figure 2:**
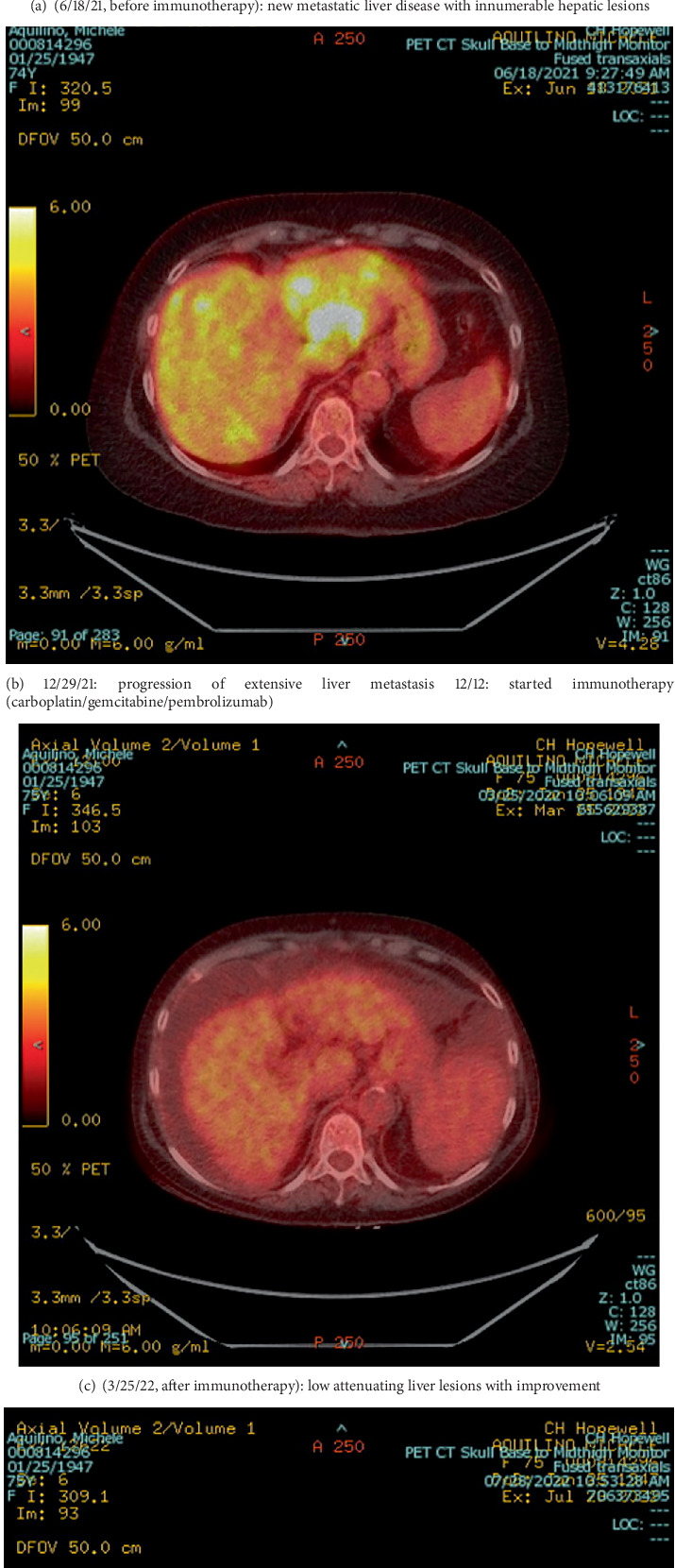
(a–d) Follow-up PET scan imaging of liver metastasis lesions at different stages of treatment. FDG, F-fluorodeoxyglucose.

**Figure 3:**
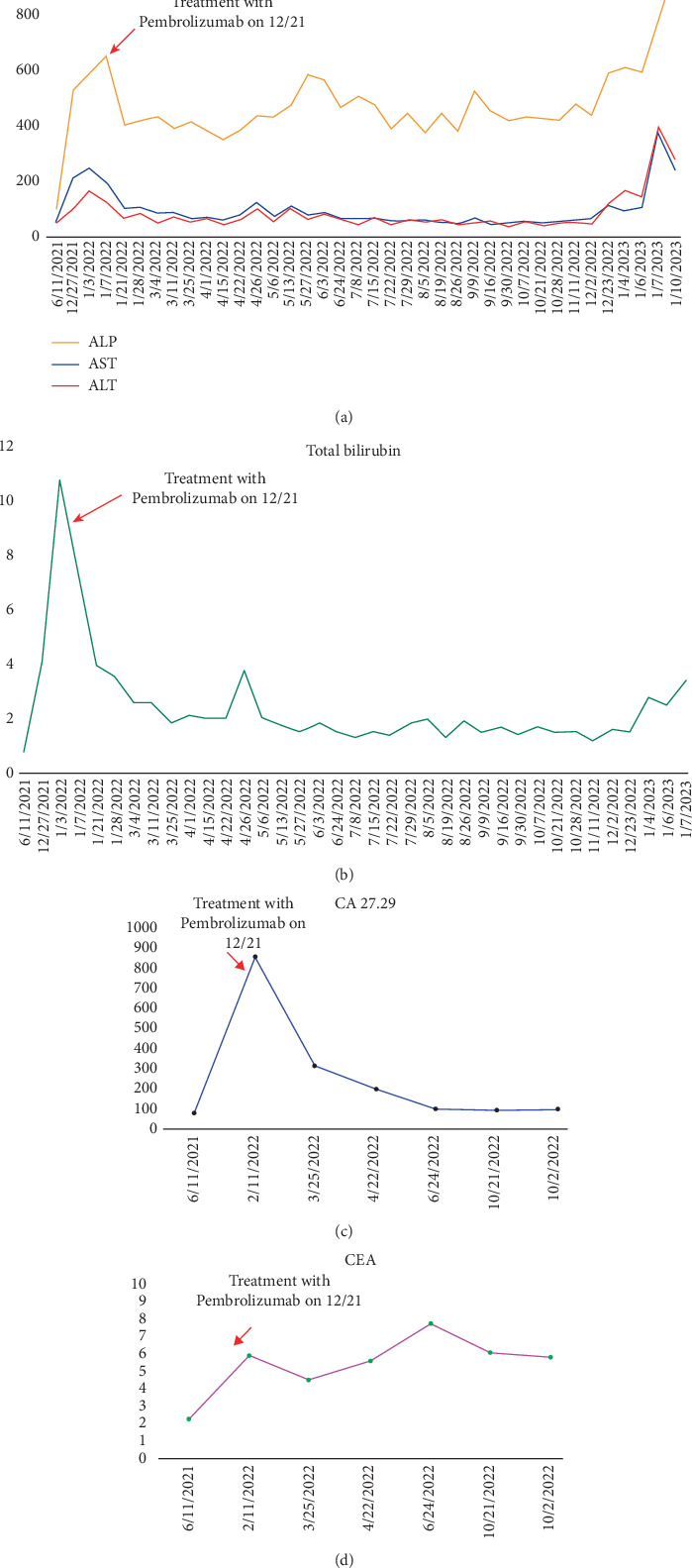
Line graphs show the trend of tumor hepatobiliary function and tumor markers. (a, b) LFTS, liver transaminases, ALP, and total bilirubin, which trended downward after starting immunotherapy. Tumor markers (c) CA 27.29 and (d) CEA. CA 27.29 dropped with pembrolizumab treatment; CEA levels also declined. ALP, alkaline phosphatase; CA, cancer antigen; CEA, carcinoembryonic antigen.

She was initially treated with fulvestrant and palbociclib, but her disease progressed, with worsening liver function tests and imaging findings. In view of her high PD-L1 expression, therapy was switched to carboplatin, gemcitabine, and pembrolizumab. Within 3 months, her liver function normalized, and imaging demonstrated a dramatic radiological response (Figures 2a, 2b, 2c, 2d, and 3).

The patient remained disease-free for approximately 10 months. Thereafter, she developed repeated hospitalizations due to infections and complications of pseudocirrhosis. Her disease subsequently progressed rapidly, and she transitioned to hospice care. She passed away shortly thereafter.

This case emphasizes the challenges of managing advanced breast cancer, while highlighting the potential benefit of immunotherapy in patients with exceptionally high PD-L1 expression, even in the ER+/HER2− subtype.

## 3. Discussion

TNBC is an aggressive and immunogenic subtype of breast cancer with a high propensity for metastasis and limited treatment options due to its lack of hormone receptors and HER2 amplification [9, 15]. Historically, chemotherapy has been the mainstay of treatment [2, 15, 16]. However, the advent of ICIs has significantly altered the therapeutic landscape, demonstrating survival benefits in appropriately selected patients [10, 11, 17–21]. This is probably because of the tumor's immunogenicity, a high proportion of tumor-infiltrating lymphocytes (TILs), tumor mutational burden, and PD-L1 expression [7, 9, 22–26]. In contrast, the role of immunotherapy in HR+ breast cancer remains under active investigation.

The first signal of activity for ICIs in HR+ breast cancer came from KEYNOTE-028, a Phase Ib multicohort basket study of pembrolizumab in PD-L1-positive advanced solid tumors. The HR+/HER2− breast cancer cohort included 25 heavily pretreated patients. While the objective response rate was modest at 12% (3 partial responses), the responses were durable with a median duration of response of ~12 months. Importantly, the trial confirmed that pembrolizumab was safe and tolerable in this population, providing early proof-of-concept that immune checkpoint inhibition can achieve disease control in select ER+ patients [27].

In the neoadjuvant setting, the I-SPY2 adaptive platform trial provided stronger evidence of benefit. This trial continuously evaluates novel agents in high-risk Stage II/III breast cancer using biomarker-driven adaptive randomization. In the ER+/HER2− high-risk cohort, the addition of pembrolizumab to standard chemotherapy nearly doubled pathologic complete response (pCR) rates to 30% compared with 13% for chemotherapy alone [28]. Although I-SPY2 is exploratory in nature, this result was important because it showed that immunotherapy could meaningfully enhance chemotherapy efficacy even in a traditionally immune-cold subtype, and it directly informed the design of Phase III trials such as KEYNOTE-756 [14].

The most definitive evidence to date comes from the CheckMate-7FL Phase III trial, which randomized 510 patients with early-stage, high-risk ER+/HER2− breast cancer to receive neoadjuvant chemotherapy with either nivolumab or placebo. The addition of nivolumab significantly improved pCR rates (24.5% vs. 13.8%, *p* = 0.0021), with the greatest benefit observed in PD-L1-positive tumors (44.3% vs. 20.2%). Safety was manageable, consistent with prior ICI experience, and while event-free survival data remain immature, the trial confirms that PD-1 blockade can potentiate chemotherapy response in this subtype [29].

In the metastatic setting, the PACE trial explored whether immunotherapy could overcome endocrine resistance after CDK4/6 inhibitor failure. Patients with HR+/HER2− metastatic breast cancer were randomized to fulvestrant alone, fulvestrant plus palbociclib, or fulvestrant plus palbociclib and the PD-L1 inhibitor avelumab [13]. While the trial did not meet its primary endpoint of improved progression-free survival, exploratory analysis revealed that the triplet arm achieved a longer median overall survival of 42.5 months compared with 27.5 months for fulvestrant alone and 24.6 months for fulvestrant plus palbociclib. These findings suggest that checkpoint blockade may synergize with endocrine and targeted therapy in a subset of patients, supporting continued investigation into rational combinations in advanced HR+ disease [30–32].

The most compelling data come from the KEYNOTE-756 trial, a randomized Phase III study enrolling 1278 patients with high-risk, Grade 3, early-stage ER+/HER2− breast cancer [14]. Patients were assigned to neoadjuvant chemotherapy with either pembrolizumab or placebo, followed by surgery, then adjuvant pembrolizumab or placebo plus endocrine therapy. Dual primary endpoints are pCR rate and EFS. The results revealed a significantly higher pCR in the pembrolizumab group compared to the control group (24.3% vs. 15.6%, *p* = 0.00005), reflecting an absolute improvement of 8.7%. These findings suggest that incorporating pembrolizumab may enhance the effectiveness of standard therapies. The study also assessed residual cancer burden (RCB) outcomes as a secondary endpoint, providing insights into the extent of residual disease posttreatment. Pembrolizumab was also associated with lower residual RCB scores, suggesting deeper responses. While EFS data remain immature, early trends favor pembrolizumab, suggesting that the addition of PD-1 blockade not only increases the chance of achieving pCR but may also translate into long-term survival benefit. Together with CheckMate-7FL, KEYNOTE-756 establishes proof-of-principle that immune checkpoint inhibition can improve outcomes in high-risk ER+/HER2− breast cancer (Table 1) [33–35].

Several published case reports mirror our patient's outcome and provide important clinical context. Wang et al. described an ER+/HER2−, TMB-high patient with a partial response to camrelizumab plus vinorelbine [36]. Wu et al. reported two ER+ cases who achieved progression-free survival beyond 21 months with pembrolizumab combined with letrozole or tamoxifen [37]. Li et al. documented an exceptional response to pembrolizumab and trastuzumab in a heavily pretreated HER2-positive patient with MSI-H and TMB-high disease [38]. Fitzpatrick and Cobleigh described a 72-year-old with lobular carcinoma and high TMB who achieved a complete response to pembrolizumab [39]. Kaplan et al. provided compelling evidence that the MLH1 1835del3 germline variant was pathogenic, with the affected patient experiencing a > 5-year remission after just four doses of pembrolizumab [40]. Collectively, these cases emphasize that rare, biomarker-defined subsets of ER+ breast cancer may achieve durable and exceptional responses to immunotherapy.

Our case adds to this growing body of evidence by demonstrating a dramatic response to pembrolizumab in HR+ metastatic breast cancer with extensive visceral involvement. Importantly, the patient's biomarker profile aligned with emerging predictors of ICI sensitivity, reinforcing the role of comprehensive molecular testing in treatment selection. This observation is consistent with recent reviews emphasizing that, although immunotherapy is not standard for HR+ breast cancer, biomarker-driven approaches and rational combinations may expand its therapeutic potential.

## 4. Conclusion

This case underlines the potential role of pembrolizumab in a select subgroup of HR+/HER2− metastatic breast cancer patients with high PD-L1 expression. The patient's durable response reinforces the need for further studies to refine predictive biomarkers and optimize immunotherapy strategies in this traditionally immunotherapy-resistant subtype. While ICIs have shown significant success in TNBC, their efficacy in HR+ breast cancer has been limited. However, emerging data suggest their potential benefit in specific subsets of patients. The PACE trial demonstrated a prolonged PFS with the addition of avelumab, and the KEYNOTE-756 trial reported a statistically significant improvement in pCR rates in the pembrolizumab arm. These findings highlight the promise of ICIs in HR+ breast tumors. Further research is essential to identify predictive biomarkers and develop personalized immunotherapy-based strategies for women with advanced breast cancer, especially those traditionally considered nonimmunogenic.

## Figures and Tables

**Table 1:** Summary of the primary studies proving clinical benefit of immunotherapy in TNBC and HR+ breast cancer.

**Trial**	**Method**	**Number of participants**	**Endnote**	**Conclusion**
KEYNOTE-028 Pembrolizumab monotherapy in PD-L1+ ER+/HER2− advanced breast cancer, post standard therapy	Phase Ib, nonrandomized, open label, multicohort study	25 (breast cancer)	Primary endpoints: Safety and ORR	ORR was 12% (three patients), all partial responses; median duration of response ~12 months; well tolerated [27]
I-SPY2 trial Pembrolizumab + neoadjuvant chemo in high-risk Stage II/III breast cancer (ER+/HER2− subset analyzed)	Phase II, adaptive randomized platform trial	~40 ER+/HER2− patients in pembrolizumab arm	Primary endpoints: pCR	Pembrolizumab + chemo nearly doubled pCR in HR+/HER2− (30% vs. 13%); strongest benefit seen in biomarker-defined high-risk cohort [28]
CheckMate-7FL Nivolumab + neoadjuvant chemo in early-stage, high-risk ER+/HER2− breast cancer	Phase III, randomized, double-blind	510 patients (257 nivolumab and 253 placebo)	Primary endpoints: pCR	Nivolumab + chemo improved pCR 24.5% vs. 13.8% (*p* = 0.0021); benefit greatest in PD-L1+ tumors (44.4% vs. 20.2%) [29]
PACE trial Adding avelumab to fulvestrant±palbociclib in HR+/HER2− MBC progressed after CDK4/6i	Multicenter, Phase 2 (three arms) • Fulvestrant • Fulvestrant + palbociclib • Fulvestrant + palbociclib + avelumab	220	The primary endpoint: PFS (fulvestrant + palbociclib vs. fulvestrant alone). Secondary endpoint: ORR, PFS for other arms	Median OS: fulvestrant 27.5 months; fulvestrant + palbociclib 24.6 months; fulvestrant + palbociclib + avelumab 42.5 months. HR for OS did not favor palbociclib alone but favored the triplet at 8.1 months [30–32]
KEYNOTE-756 Pembrolizumab + neoadjuvant chemo, then adjuvant pembrolizumab + endocrine therapy in HR+/HER2− breast cancer	Phase III, randomized, double-blind	1278	Dual primary end points: pCR and EFS	Pembrolizumab in the neoadjuvant setting improved pCR rates in ER+/HER2− breast cancer 24.3% vs. 15.6% (14) [33–35]

## Data Availability

The data that support the findings of this study are available on request from the corresponding author. The data are not publicly available due to privacy or ethical restrictions.

## Nomenclature

ER+: estrogen receptor-positive
PR−: progesterone receptor-negative
HER2−: human epidermal growth factor receptor 2-negative
TNBC: triple-negative breast cancer
ICIs: immune checkpoint inhibitors
PD-L1: programmed death-ligand 1
CPS: combined positive score
TILs: tumor-infiltrating lymphocytes
MRM: modified radical mastectomy
PET: positron emission tomography
ddAC-Taxol: dose-dense adriamycin/cyclophosphamide followed by taxol
GATA3: GATA binding protein 3
IHC: immunohistochemistry
CDK4/6: cyclin-dependent kinase 4/6
PFS: progression-free survival
OS: overall survival
pCR: pathologic complete response
EFS: event-free survival
RCB: residual cancer burden
ALP: alkaline phosphatase
CA 27.29: cancer antigen 27.29
CEA: carcinoembryonic antigen
FDA: U.S. Food and Drug Administration

## References

[1] American Cancer Society (2024). Breast Cancer Facts & Figures 2024. *Cancer.org*.

[2] Bleicher R. J. (2018). Timing and Delays in Breast Cancer Evaluation and Treatment. *Annals of Surgical Oncology*.

[3] National Cancer Institute Cancer Stat Facts: Female Breast Cancer Subtypes. https://seer.cancer.gov/statfacts/html/breast-subtypes.html.

[4] Fasching P. A., Kreipe H., Del Mastro L. (2024). Identification of Patients With Early HR+ HER2- Breast Cancer at High Risk of Recurrence. *Geburtshilfe und Frauenheilkunde*.

[5] Clarke R., Tyson J. J., Dixon J. M. (2015). Endocrine Resistance in Breast Cancer--An Overview and Update. *Molecular and Cellular Endocrinology*.

[6] Ma J., Chan J. J., Toh C. H., Yap Y. S. (2023). Emerging Systemic Therapy Options Beyond CDK4/6 Inhibitors for Hormone Receptor-Positive HER2-Negative Advanced Breast Cancer. *npj Breast Cancer*.

[7] Núñez Abad M., Calabuig-Fariñas S., Lobo de Mena M. (2022). Programmed Death-Ligand 1 (PD-L1) as Immunotherapy Biomarker in Breast Cancer. *Cancers*.

[8] Liu Y., Hu Y., Xue J. (2023). Advances in Immunotherapy for Triple-Negative Breast Cancer. *Molecular Cancer*.

[9] Goff S. L., Danforth D. N. (2021). The Role of Immune Cells in Breast Tissue and Immunotherapy for the Treatment of Breast Cancer. *Clinical Breast Cancer*.

[10] Planes-Laine G., Rochigneux P., Bertucci F. (2019). PD-1/PD-L1 Targeting in Breast Cancer: The First Clinical Evidences Are Emerging. A Literature Review. *Cancers*.

[11] de Mello R. A., Amaral G. A., Tajima C. C. (2021). Potential Role of Immunotherapy for Advanced Breast Cancer. *Immunotherapy*.

[12] Goldberg J., Pastorello R. G., Vallius T. (2021). The Immunology of Hormone Receptor Positive Breast Cancer. *Frontiers in Immunology*.

[13] Mayer E. L., Ren Y., Wagle N. (2024). PACE: A Randomized Phase II Study of Fulvestrant, Palbociclib, and Avelumab After Progression on Cyclin-Dependent Kinase 4/6 Inhibitor and Aromatase Inhibitor for Hormone Receptor-Positive/Human Epidermal Growth Factor Receptor-Negative Metastatic Breast Cancer. *Journal of Clinical Oncology: Official Journal of the American Society of Clinical Oncology*.

[14] Cardoso F., O'Shaughnessy J., Liu Z. (2025). Pembrolizumab and Chemotherapy in High-Risk, Early-Stage, ER+/HER2- Breast Cancer: A Randomized Phase 3 Trial. *Nature Medicine*.

[15] Aysola K., Desai A., Welch C. (2013). Triple Negative Breast Cancer-An Overview. *Hereditary Genetics: Current Research*.

[16] Foulkes W. D., Smith I. E., Reis-Filho J. S. (2010). Triple-Negative Breast Cancer. *The New England Journal of Medicine*.

[17] Chen F., Chen N., Gao Y., Jia L., Lyu Z., Cui J. (2022). Clinical Progress of PD-1/L1 Inhibitors in Breast Cancer Immunotherapy. *Frontiers in Oncology*.

[18] Howard F. M., Villamar D., He G., Pearson A. T., Nanda R. (2022). The Emerging Role of Immune Checkpoint Inhibitors for the Treatment of Breast Cancer. *Expert Opinion on Investigational Drugs*.

[19] Emens L. A. (2018). Breast Cancer Immunotherapy: Facts and Hopes. *Clinical Cancer Research: An Official Journal of the American Association for Cancer Research*.

[20] Emens L. A., Ascierto P. A., Darcy P. K. (2017). Cancer Immunotherapy: Opportunities and Challenges in the Rapidly Evolving Clinical Landscape. *European Journal of Cancer*.

[21] Loison R., Loirat D. (2022). Pembrolizumab – Cancer du Sein TN Métastatique PD-L1 Positif. *Bulletin du Cancer*.

[22] O'Meara T., Marczyk M., Qing T. (2020). Immunological Differences Between Immune-Rich Estrogen Receptor-Positive and Immune-Rich Triple-Negative Breast Cancers. *JCO Precision Oncology*.

[23] D'Amico P., Cristofanilli M. (2022). Standard of Care in Hormone Receptor-Positive Metastatic Breast Cancer: Can We Improve the Current Regimens or Develop Better Selection Tools?. *JCO Oncology Practice*.

[24] Patel K. K., Hassan D., Nair S. (2022). Role of Immunotherapy in the Treatment of Triple-Negative Breast Cancer: A Literature Review. *Cureus*.

[25] Hargadon K. M., Johnson C. E., Williams C. J. (2018). Immune Checkpoint Blockade Therapy for Cancer: An Overview of FDA-Approved Immune Checkpoint Inhibitors. *International Immunopharmacology*.

[26] Angelico G., Broggi G., Tinnirello G. (2023). Tumor Infiltrating Lymphocytes (TILS) and PD-L1 Expression in Breast Cancer: A Review of Current Evidence and Prognostic Implications From Pathologist's Perspective. *Cancers*.

[27] Rugo H. S., Delord J. P., Im S. A. (2018). Safety and Antitumor Activity of Pembrolizumab in Patients With Estrogen Receptor-Positive/Human Epidermal Growth Factor Receptor 2-Negative Advanced Breast Cancer. *Clinical Cancer Research: An Official Journal of the American Association for Cancer Research*.

[28] Huppert L. A., Wolf D., Yau C. (2025). Pathologic Complete Response (pCR) Rates for Patients With HR+/HER2- High-Risk, Early-Stage Breast Cancer (EBC) by Clinical and Molecular Features in the Phase II I-SPY2 Clinical Trial. *Annals of Oncology*.

[29] Loi S., McArthur H. L., Harbeck N. (2020). A Phase III Trial of Nivolumab With Neoadjuvant Chemotherapy and Adjuvant Endocrine Therapy in ER+/HER2− Primary Breast Cancer: CheckMate 7FL. *Journal of Clinical Oncology*.

[30] Bross P. F., Cohen M. H., Williams G. A., Pazdur R. (2002). FDA Drug Approval Summaries: Fulvestrant. *The Oncologist*.

[31] Goodman A. (2023). *Palbociclib/Fulvestrant Does Not Improve Progression-Free Survival After Progression on a CDK4/6 Inhibitor in Metastatic Breast Cancer*.

[32] Mayer E. L., Ren Y., Wagle N. (2023). Abstract GS3-06: GS3-06 Palbociclib After CDK4/6i and Endocrine Therapy (PACE): A Randomized Phase II Study of Fulvestrant, Palbociclib, and Avelumab for Endocrine Pre-treated ER+/HER2- Metastatic Breast Cancer. *Cancer Research*.

[33] Cardoso F., McArthur H. L., Schmid P. (2023). LBA21 KEYNOTE-756: Phase III Study of Neoadjuvant Pembrolizumab (Pembro) or Placebo (Pbo) + Chemotherapy (Chemo), Followed by Adjuvant Pembro or Pbo + Endocrine Therapy (ET) for Early-Stage High-Risk ER+/HER2– Breast Cancer. *Annals of Oncology*.

[34] Monberg M. J., Keefe S., Karantza V. (2024). A Narrative Review of the Clinical, Humanistic, and Economic Value of Pembrolizumab-Based Immunotherapy for the Treatment of Breast and Gynecologic Cancers. *Oncology and Therapy*.

[35] Helwick C. (2024). *KEYNOTE-756 Additional Findings: Pembrolizumab Shown to Be Beneficial Regardless of Age, Menopausal Status*.

[36] Wang R., Yang Y., Ye W. W. (2021). Case Report: Significant Response to Immune Checkpoint Inhibitor Camrelizumab in a Heavily Pretreated Advanced ER+/HER2- Breast Cancer Patient With High Tumor Mutational Burden. *Frontiers in Oncology*.

[37] Wu D., Tang S., Ye R. (2021). Case Report: Long-Term Response to Pembrolizumab Combined With Endocrine Therapy in Metastatic Breast Cancer Patients With Hormone Receptor Expression. *Frontiers in Immunology*.

[38] Li A., Goodyear S., Fuss C., Mitri Z. (2021). Exceptional Response to Pembrolizumab and Trastuzumab in a Heavily Pretreated Patient With HER2-Positive TMB-H and MSI-H Metastatic Breast Cancer. *JCO Precision Oncology*.

[39] Fitzpatrick R., Cobleigh M. (2020). Extreme Response to Immunotherapy in an Estrogen Receptor Positive Breast Cancer: A Case Report. *Clinical Oncology: Case Reports*.

[40] Kaplan H. G., Whiteaker J. R., Nelson B. (2023). Hormone Receptor-Positive Breast Cancer Sensitive to Pembrolizumab: Evidence of the Pathogenicity of the *MLH1* Variant 1835del3. *Journal of the National Comprehensive Cancer Network*.

